# Quality assurance analysis of hippocampal avoidance in a melanoma whole brain radiotherapy randomized trial shows good compliance

**DOI:** 10.1186/s13014-018-1077-z

**Published:** 2018-07-20

**Authors:** Geoffrey Martinage, Angela M. Hong, Mike Fay, Thanuja Thachil, Daniel Roos, Narelle Williams, Serigne Lo, Gerald Fogarty

**Affiliations:** 1 Melanoma Institute Australia, The University of Sydney, NSW North Sydney, Australia; 2 Centre Oscar-Lambret, Lille, France; 3 Mater Hospital, NSW North Sydney, Australia; 4 GenesisCare, Radiation Oncology, Mater Hospital, NSW North Sydney, Australia; 5 Central Clinical School, The University of Sydney, Camperdown, NSW Australia; 6 School of Medicine and Public Health, University of Newcastle, NSW Callaghan, Australia; 7 GenesisCare, Radiation Oncology, NSW Newcastle, Australia; 8 Northern Territory Radiation Oncology, Alan Walker Cancer Care Centre, NT Darwin, Australia; 9 Royal Adelaide Hospital, Adelaide, South Australia Australia; 10 University of Adelaide, South Australia Adelaide, Australia; 11 Australia and New Zealand Melanoma Trials Group, NSW North Sydney, Australia

**Keywords:** Radiotherapy, Whole brain radiotherapy, Trial, Quality assurance, Intensity-modulated radiotherapy, Hippocampal avoidance

## Abstract

**Background:**

Melanoma brain metastases (MBM) often cause morbidity and mortality for stage IV melanoma patients. An ongoing randomised phase III trial (NCT01503827 – WBRT-Mel) evaluates the role of adjuvant whole brain radiotherapy (WBRT) following local treatment of MBM. Hippocampal avoidance during WBRT (HA-WBRT) has shown memory and neurocognitive function (NCF) preservation in the RTOG-0933 phase II study. This study assessed the quality assurance of HA-WBRT within the WBRT-Mel trial according to RTOG-0933 study criteria.

**Methods:**

Hippocampal avoidance was allowed in approved centres with intensity-modulated radiotherapy capability. Patients treated by HA-WBRT were not randomized within the WBRT arm. The RTOG 0933 contouring Atlas was used to contour hippocampi. In the trial co-ordinating centre, patients were treated with volumetric modulated arc therapy using complementary arcs; similar techniques were used at other sites. Dosimetric data were extracted retrospectively and analysed in accordance with RTOG 0933 study constraints criteria.

**Results:**

Among the 215 patients accrued to the WBRT-Mel study between April 2009 and September 2017, 107 were randomized to the WBRT arm, 22 were treated by HA-WBRT in 4 centers. Eighteen patients were treated in the same centre. The median age was 65 years. The commonest (91%) HA-WBRT schema was 30 Gy in 10 fractions. Prior to HA-WBRT, 10 patients had been treated by surgery alone, six by radiosurgery alone, four by surgery and radiosurgery and two exclusively by simultaneous integrated boost concurrent to HA-WBRT. Twenty patients were treated with intention to spare both hippocampi and two patients had MBM close to one hippocampus and were treated with intention to spare the contralateral hippocampus. According to RTOG-0933 study criteria, 18 patients (82%) were treated within constraints and four patients (18%) had unacceptable deviation in just one hippocampus.

**Conclusions:**

This dosimetric quality assurance study shows good compliance (82%) according to RTOG-0933 study dosimetric constraints. Indeed, all patients respected RTOG hippocampal avoidance constraints on at least one hippocampus. In the futureanalysis of the WBRT-Mel trial, the NCF of patients on the observation arm, WBRT arm and with HA-WBRT arm will be compared.

## Background

Melanoma brain metastases (MBM) often cause morbidity and mortality for patients with stage 4 melanoma [1–7]. The treatment landscape has changed recently with effective systemic therapy that can cross the blood brain barrier [8–11], new surgery and radiotherapy treatment techniques, such as better minimally invasive surgery, stereotactic radiosurgery and intensity-modulated radiotherapy (IMRT)*.* As a result, these patients are now living longer, often requiring repeated interventions for MBM. Consequently the long-term effects of radiotherapy treatment on and neurocognitive function and quality of life have become even more important [12, 13].

Prior to this changing landscape, a phase III randomized trial (RCT) was started in 2009 to compare whole brain radiotherapy (WBRT) with observation following local treatment (surgery or radiosurgery) of 1–3 MBM (ClinicalTrials.gov identifier: NCT01503827 – WBRT-Mel trial). The primary endpoint of the trial was 12-month intracranial control, with secondary endpoints including neurocognitive function (NCF) and quality of life. Inclusion criteria have been previously detailed [14]. Previous data had shown that cells of the hippocampus are especially sensitive to even low doses of radiation [15]. Concurrently, it was found that the hippocampus in oligometastatic disease was relatively spared from metastasis [16–18] and the IMRT technique has been developed to spare the hippocampus during WBRT to preserve the NCF [19–21]. An RTOG phase II trial showed that hippocampal avoidance during WBRT (HA-WBRT) could minimise the neurocognitive decline at six  months compared to historical controls [13]. With these new data, the WBRT-Mel protocol was modified in 2013 to allow HA-WBRT for those randomized to the WBRT arm. The WBRT-Mel completed its accrual of 215 patients in September 2017. The plan is to analyse the NCF endpoints in the three treatment cohorts: observation, HA-WBRT and non HA-WBRT.

The validity of the results from this cohort depend on whether the hippocampi of these patients have been spared according to the RTOG 0933 phase II trial [13]. These dosimetric criteria for hippocampal sparing from quality assurance of the RTOG-0933 study are summarized in Table 1 [22]. The most important criteria in this Table 1 for the quality assurance of hippocampal avoidance are D100% and Dmax for each hippocampus. Dx% and Vy represent respectively the dose received by x % and the volume received by y Gy of specified structure. This study reports the radiotherapy quality assurance of patients treated with HA-WBRT on the WBRT-Mel trial.

**Table 1:** HA-WBRT Planning acceptability defined by RTOG 0933

	Parameter	Per Protocol	Acceptable Variation	Unacceptable Deviation
HA-WBRT IMRT Planning	PTV	D2% ≤ 37.5 Gy D98% ≥ 25 Gy	37.5Gy < D2% ≤ 40Gy D98% < 25 Gy	V30 < 90% D2% > 40 Gy
	Hippocampus	D100% ≤ 9 Gy Dmax ≤16Gy	D100% ≤ 10 Gy Dmax ≤17 Gy	D100% > 10 Gy Dmax > 17 Gy
	Optic nerves and chiasm	Dmax ≤37.5Gy	Dmax ≤37.5Gy	Dmax > 37.5Gy
Unscheduled Break Days		0 break days	1–3 break days	> 3 break days

*Dmax* Maximum dose, *HA-WBRT* Hippocampal avoidance during whole brain radiotherapy, *IMRT* Intensity-modulated radiotherapy, *PTV* Planning target volume [13, 22]. Dx% and Vy represent respectively the dose received by x % and the volume received by y Gy of specified structure

## Methods

### Population

The WBRT-Mel Trial and protocol amendment to include HA-WBRT were approved by relevant ethics committees and the data safety monitoring committee. Patients treated by HA-WBRT were stratified but not randomized within the WBRT arm of the trial.

### HA-WBRT planning technique

Patients were immobilised with a thermoplastic mask in a neutral head position. A non-contrast planning CT was acquired at 1 mm slice thickness and fused with the diagnostic MRI scan. The radiation oncologist contoured the right and left hippocampi on the fused MRI-CT image set with T1-weighted MRI axial sequences using the contouring Atlas of the RTOG 0933 trial [23]. Hippocampal avoidance regions were generated by three-dimensionally expanding the hippocampal contours by 5 mm. The planning target volume (PTV) was defined as the whole-brain parenchyma excluding the hippocampal avoidance regions [22]. Other organs at risk (optic nerves, chiasm, eyes and lenses) were contoured. Patients were planned and treated on different platforms. In the trial co-ordinating center, patients were planned with Eclipse (version 11.0.47) radiotherapy treatment planning system (Varian), treated with a Varian 21iX linear accelerator (RapidArc) using two complementary arcs.

### Statistical analysis

Data collected included hippocampal volumes, hippocampal maximum dose, hippocampal minimum dose (D100%), Hippocampus volume receiving 10 Gy (V10Gy) and dose to 40 and 50% of the hippocampus (D40% and D50%, respectively). For patients with another schema of treatment other than 30 Gy in 10 fractions, the equivalent dose assuming an α/β ratio of 2.0 for hippocampus was calculated [24] by iLQ (v2.0). All volumes measured at the patient level were summarized by their median (range) and stratified by whether or not patients had unacceptable deviation (UD) or not, according to RTOG 0933 constraints criteria. Volume difference between the two groups UD versus no UD was tested through the Wilcoxon rank test. All tests were two sided with a nominal *p* value of 0.05.

## Results

### Patient characteristics

Among the 215 patients accrued to the WBRT-Mel study between April 2009 and September 2017, a total of 107 patients were randomized to the WBRT arm and 22 of them were treated with HA-WBRT. The median age of these 22 patients was 65 years at randomisation (range 27–88, Table 2). Prior to HA-WBRT, 10 patients had been treated by surgery only, six by stereotactic radiosurgery (SRS) only, four by surgery and SRS (two for the same lesion and two for another metastasis) and two by simultaneous integrated boost (SIB) concurrent with HA-WBRT. SIB during HA-WBRT was performed for six patients (3 to untreated lesions only, 1 to surgical cavity only and 2 to untreated lesions and surgical cavity).

**Table 2:** Patient and volume characteristics

	HA-WBRT (*n* = 22)	Bilateral HA-WBRT (*n* = 20)	Unilateral (right) HA-WBRT (*n* = 2)
Female	5 (23%)	5 (25%)	0
Male	17 (77%)	15 (75%)	2 (100%)
Median age (years)	65 (27–88)	65 (27–88)	69 (61–76)
≤ 65 years	11 (50%)	10 (50%)	1 (50%)
> 65 years	11 (50%)	10 (50%)	1 (50%)
Number of MBM:			
1 MBM	13 (59%)	12 (60%)	1 (50%)
2 MBM	7 (32%)	6 (30%)	1 (50%)
3 MBM	2 (9%)	2 (10%)	0
First treatment of MBM:			
Surgery only	10 (45%)	9 (45%)	1 (50%)
SRS only	6 (27%)	5 (25%)	1 (50%)
Surgery and SRS	4 (18%)	4 (20%)	0
Exclusive SIB concurrent with HA-WBRT	2 (9%)	2 (10%)	0
SRS:			
Median volume (cm^3^)	2.07 (0.04–42.6)	3.01 (0.04–42.6)	0.96 (0.96)
Median dose (Gy)	20 (14–22)	20 (14–22)	16 (16)
Volumes (cm^3^):			
PTV volume	1434.8	1412.7	1516.9
Median (Range)	(1177–1640)	(1177–1640)	(1488–1545)
R hippocampus volume	2.27	2.27	2.54
Median (Range)	(0.9–5.66)	(0.90–5.66)	(1.80–3.27)
L hippocampus volume	2.02	1.96	2.58
Median (Range)	(1.01–4.45)	(1.01–4.45)	(2.58)
Volume of 2 hippocampus	4.47	4.38	5.85
Median (Range)	(2.28–9.98)	(2.28–9.98)	(5.85)
Sparing volume of 2 hippocampus	29.52	28.53	34.77
Median (Range)	(13.4–49.4)	(13.4–49.4)	(34.7)
Details of the SIB treatment:	*n* = 6	*n* = 5	*n* = 1
1 MBM	3 (50%)	3 (60%)	0
2 MBM	2 (33%)	1 (20%)	1
3 MBM	1 (17%)	1 (20%)	0
Median volume (cm^3^)	5.71 (0.54–29.9)	5.71 (0.74–29.9)	6.26 (0.54–11.97)
Median dose (Gy)	49.5 (40–50.4)	47.3 (40–50.4)	50 (50)
Median distance to nearest hippocampus (cm)	2.8 (0–5.0)	3.8 (2.0–5.0)	0.3 (0–0.6)

Bilateral/Unilateral HA-WBRT: Patients treated with intention to spare bilateral/unilateral hippocampus, *HA-WBRT* Hippocampal avoidance during whole brain radiotherapy, *Hippo* Hippocampus, *MBM* Melanoma brain metastases, *PTV* Planning target volume, *SIB* Simultaneous integrated boost, *SRS* Stereotactic radiosurgery, *WBRT* Whole brain radiotherapy

HA-WBRT was delivered in four centers. Eighteen patients (82%) were enrolled by the Melanoma Institute Australia and treated at the Mater hospital, GenesisCare. The commonest fractionation of adjuvant WBRT was 30 Gy in 10 fractions, delivered to 20 patients (91%). One patient was treated to 30 Gy in 12 fractions with three SIB areas to 50.4 Gy. The second patient was treated to a whole brain dose of 32.4 Gy in 15 fractions with SIB area to 49.5 Gy.

**Fig. 1:**
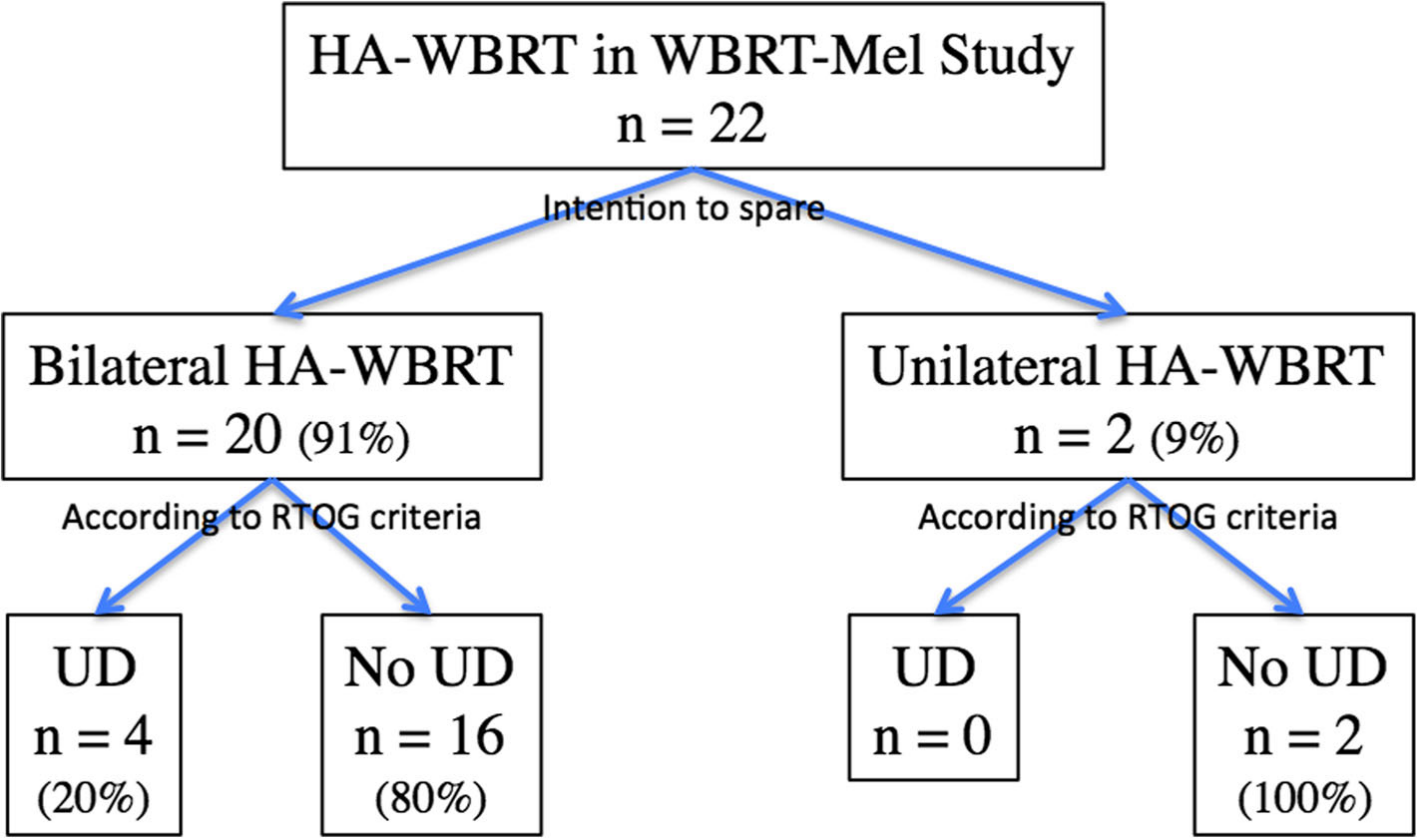
QA HA-WBRTMel Study Flowchart. HA-WBRT: Hippocampal Avoidance during Whole Brain Radiotherapy; QA: Quality Assurance; UD: Unacceptable Deviation

Twenty patients (91%) were treated with intention to spare both hippocampi and two patients (9%) had MBM very close to the hippocampus (0 and 6.5 mm from the left hippocampus) and were treated with intention to spare the contralateral hippocampus only (Fig. 1). One patient had MBM inside the left hippocampus treated by SRS and the second had MBM of 0.54 cm^3^ inside a 6.5 mm margin around the left hippocampus, treated by SIB (50 Gy in 10 fractions).

### Critical structure constraints analysis according to RTOG 0933 criteria

According to RTOG constraints criteria, 22 (100%) were within hippocampal avoidance constraints on a minimum of one hippocampus. Eighteen patients (81%) respected these constraints and four patients (19%) respected them on one hippocampus. Characteristics of patients and patient volumes with and without UD are compared in Table 3. At baseline, patients’ characteristics were similar, however patients with UD had significantly greater left hippocampal volume, total hippocampi volume and sparing volume of left hippocampus than patients without UD. The summary of acceptable variation (AV) and UD are presented in Table 4. Four patients (18%) had UD on one hippocampus and their treatment plans are detailed in Table 5. The two first patients with UD on one hippocampus were treated before the results of RTOG 0933. The third patient had hippocampus Dmax of 17.24 Gy and the fourth patient had hippocampus D100% of 10.33 Gy.

**Table 3:** Patient, treatment and volume characteristics in patients with and without unacceptable deviation (UD)

Variable	Patients without UD (*n* = 18)	Patient with UD (*n* = 4)	*p*-value*
Gender			
Female	4/18 (22%)	1/4 (25%)	1.000
Male	14/18 (78%)	3/4 (75%)	1.000
Median age at randomization (range)	68 (37–88)	48 (27–73)	0.121
Surgery			
No	6/18 (33%)	2/4 (50%)	0.602
Yes	12/18 (67%)	2/4 (50%)	
Number of lesions treated by surgery			
1	11/12 (92%)	1/2 (50%)	
2	1/12 (8%)	1/2 (50%)	
SRS			
No	10/18 (56%)	2/4 (50%)	1.000
Yes	8/18 (44%)	2/4 (50%)	1.000
Number of lesions treated by SRS			
1	6/8 (75%)	1/2 (50%)	
2	2/8 (25%)	1/2 (50%)	
Simultaneous Integrated Boost			
No	13/18 (72%)	3/4 (75%)	1.000
Yes	5/18 (28%)	1/4 (25%)	
Number of SIB treatment			
1	2/5 (40%)	1/1 (100%)	
2	2/5 (40%)	0	
3	1/5 (20%)	0	
Right hippocampus:			
Volume (cm^3^)	2.0 (0.9–3.9)	3.8 (2.1–5.7)	0.052
Avoidance Volume (cm^3^)	13.6 (6.8–20.7)	17.0 (15.4–27.9)	0.080
Left hippocampus:			
Volume (cm^3^)	1.9 (1.0–3.3)	4.1 (2.4–4.5)	0.004
Avoidance Volume (cm^3^)	13.3 (6.7–20.3)	17.5 (15.3–21.5)	0.035
Both hippocampi:			
Volume (cm^3^)	4.0 (2.3–7.2)	7.9 (4.5–10.0)	0.013
Avoidance volume (cm^3^)	26.7 (13.5–41.0)	34.0 (31.7–49.4)	0.061

*Based on Wilcoxon rank-sum test or Fisher exact test as appropriate. *SIB* Simultaneous Integrated Boost, *SRS* Stereotactic Radiosurgery

**Table 4:** Summary of number of acceptable variation and/or unacceptable deviation according to RTOG dosimetric constraints criteria [22]

	Per protocol Number (%)	AV Number (%)	UD Number (%)	Comment
R hippocampus (*n* = 22)	16 (73%)	3 (14%)	3 (14%)	
L Hippocampus (*n* = 20)	17 (85%)	2 (10%)	1 (5%)	1 patient had AV on both hippocampi
Patients (*n* = 22)	16 (73%)	4 (18%)	4 (18%)	2 patients had UD on one hippocampus and AV on the second hippocampus
Optic nerves (*n* = 22)	22 (100%)	0	0	
Chiasm (*n* = 22)	22 (100%)	0	0	
Unscheduled break	19 (86%)	3 (14%)	0	

*AV* Acceptable variation, *L* Left, *PTV* Planning target volume, *R* Right, *UD* Unacceptable deviation

**Table 5:** Details of the dosimetric unacceptable deviations of HA-WBRT according to RTOG 0933 dosimetric constraints (22)

Patient	Hippo	UD Value	Max value	Excess over 0933 UD as percentage	Comments
1	Right	Dmax (> 17 Gy)	17.85 Gy	5.0%	RT given prior to RTOG 0933 constraints being published
2	Right	Dmin (> 10 Gy)	10.4 Gy	4.0%	RT given prior to RTOG 0933 constraints being published
3	Left	Dmax (> 17 Gy)	17.24 Gy	1.4%	Multiple dosimetry has been realized to decrease the Dmax on left hippocampus. This patient had D2%Left_hippo of 14.05 Gy
4	Right	Dmin (> 10 Gy)	10.33 Gy	3.3%	

*Dmax* Maximum dose, *Dmin* Minimum dose, *HA-WBRT* Hippocampal avoidance during whole brain radiotherapy, *Hippo* Hippocampus, *Max* Maximum, *MBM* Melanoma brain metastases, *RT* Radiotherapy, *SIB* Simultaneous integrated boost, *SRS* Radiosurgery, *UD* Unacceptable deviation (22)

The hippocampi D40% median was calculated in 2 Gy equivalent and was 7.16 Gy (range 6.2 to 9.26 Gy) with α/β ratio of 2.0 Gy for hippocampi. All patients were treated with less than 3 break days and had optic nerve/chiasm Dmax less than 37.5 Gy.

## Discussion

In this dosimetric audit of HA-WBRT of the WBRT-Mel study, all 22 patients (100%) respected RTOG 0933 hippocampal avoidance constraints of at least one hippocampus. Eighteen patients (82%) had no unacceptable deviation and of these, 16 (73%) were treated within these constraints on both hippocampi and two (9%) were planned to have sparing of one hippocampus. Four patients (18%) had unacceptable deviation on one hippocampus and none had unacceptable deviation on both hippocampi. Our results are similar to the quality assurance report of phase II RTOG 0933 trial. In the RTOG0933 trial, 82 cases were reviewed prior to treatment, 21 cases (25%) had UD. Ten cases (12%) had UD of IMRT planning, five (6%) of them had UD of both contouring and IMRT planning and 11 cases (13%) had UD of contouring only [22].

Two other smaller studies [25, 26] showed very high compliance (100%) according to RTOG constraints [22]. Some recent dosimetric studies, each with about 10 cases, investigated the dosimetric feasibility of HA-WBRT according to RTOG constraints and showed excellent compliance (100%) [27–32]. Some of them had realized a dose reduction to other organs at risk at the same time. Our compliance remains high quality despite these deviations. The first two patients treated with HA-WBRT both had UD, demonstrating a learning curve for the radiation oncologists.

It has been shown that unilateral hippocampal avoidance during WBRT can also mitigate cognitive decline, formation of memory, verbal memory, similar to bilateral HA-WBRT [33–36]. Furthermore, meta-analysis of 33 studies, which evaluated memory before and after resection of left or right anterior temporal lobe for temporal lobe epilepsy has observed difference in verbal memory function after resection from the left or right temporal lobe [37]. It is important to note that in RTOG 0933, patients with hippocampal or peri hippocampal metastases were not eligible. The trial still had 25% of cases with UD, and yet still showed a significantly superior result in terms of NCF preservation when compared to historical controls. In the light of our literature review we therefore feel comfortable in the future analysis of the NCF for the WBRT-Mel trial in assuming thatthose who had unilateral HA-WBRT will be assumed to have hippocampal avoidance adequate for NCF preservation.

There are only a few prospective evaluations of hippocampal radiation dose volume effect and memory deficit [38–41]. Currently, two phases III randomized trials are ongoing and evaluating HA-WBRT. At first, NRG Oncology-CC001 (ClinicalTrials.gov identifier: NCT02360215) assesses memantine hydrochloride and WBRT with or without hippocampal sparing technique in reducing neurocognitive decline. The second trial is NRG Oncology-CC003 (ClinicalTrials.gov identifier: NCT02635009) assessing WBRT with or without HA-WBRT in treating patients with limited stage or extensive stage small cell lung cancer. Although the RTOG 0933 study has defined hippocampal avoidance dosimetric constraints, detailed dose–volume analyses are vital in guiding the clinician in striking the balance between local tumor control and NCF preservation.

If the WBRTMel study and the other phase III study about HA-WBRT show good clinical outcomes for HA-WBRT, this technique could be more frequently used in the management of brain metastases. This change in practice could be implemented quickly as the majority of radiation oncology department have access to IMRT in developed countries.

This quality assurance study has some limitations as the limited sample size, these patients were accrued over 8.5 years and during this period, some innovation of IMRT techniques appeared (7). The hippocampi volumes were not reviewed and the Hausdorff distances were not calculated. In its favour, this quality assurance study found high reproducibility in prospective data as 82% of patients were treated in the same hospital and 73% by the same radiation oncologist.

## Conclusions

This dosimetric quality assurance study of HA-WBRT from the WBRTMel study shows good compliance (82%) according to RTOG 0933 dosimetric constraints. Indeed, all patients respected RTOG 0933 hippocampal avoidance constraints of at least one hippocampus. Eighteen patients (82%) had no unacceptable deviation, with 16 (73%) of these treated with these constraints on both hippocampi and two (9%) planned to have sparing of one hippocampus. Four patients (18%) had IMRT planning unacceptable deviation on one hippocampus. On the basis of these data, the future comparison of the NCF of patients in the observation arm, non-HA-WBRT arm and HA-WBRT will provide an accurate assessment of radiation therapy on NCF.

## Abbreviations

ANZMTG: Australia and New Zealand melanoma trials group
ASTRO: American society for radiation oncology
AV: Acceptable variation
CT: Computerized tomography
HA-WBRT: Hippocampal avoidance during whole brain radiotherapy
Hippo: Hippocampus
IMRT: Intensity-modulated radiation therapy
MBM: Melanoma brain metastases
MRI: Magnetic resonance imaging
NCF: Neurocognitive functions
PTV: Planning target volume
QA: Quality assurance
RCT: Randomized trial
RT: Radiotherapy
RTOG: Radiation therapy oncology group
SIB: Simultaneous integrated boost
SRS: Stereotactic radiosurgery
UD: Unacceptable deviation
VMAT: Volumetric modulated arc therapy
WBRT: Whole brain radiotherapy
WBRT-Mel: Whole brain radiotherapy melanoma trial

## References

[1] Barnholtz-Sloan JS, Sloan AE, Davis FG, Vigneau FD, Lai P, Sawaya RE (2004). Incidence proportions of brain metastases in patients diagnosed (1973 to 2001) in the metropolitan Detroit Cancer surveillance system. J Clin Oncol.

[2] Hong A, Fogarty G, Izard MA. The role of radiation therapy in the management of metastatic melanoma in the brain. Int J Surg Oncol [Internet]. 2012 [cited 2018 Feb 4];2012. Available from: https://www.ncbi.nlm.nih.gov/pmc/articles/PMC3332202/10.1155/2012/294735 PMC3332202 22577532

[3] Fogarty GB, Hong A, Jacobsen KD, Reisse CH, Shivalingam B, Burmeister B (2014). Accrual to a randomised trial of adjuvant whole brain radiotherapy for treatment of melanoma brain metastases is feasible. BMC Res Notes..

[4] Fogarty GB, Hong A, Dolven-Jacobsen K, Reisse CH, Burmeister B, Haydu LH (2015). First interim analysis of a randomised trial of whole brain radiotherapy in melanoma brain metastases confirms high data quality. BMC Res Notes.

[5] Taillibert S, Le Rhun É (2015). Epidemiology of brain metastases. Cancer Radiother J Soc Francaise Radiother Oncol.

[6] Fogarty GB, Hong A, Gondi V, Burmeister B, Jacobsen K, Lo S (2016). Debate: adjuvant whole brain radiotherapy or not? More data is the wiser choice. BMC Cancer.

[7] Brown PD, Ahluwalia MS, Khan OH, Asher AL, Wefel JS, Gondi V (2017). Whole-brain radiotherapy for brain metastases: evolution or revolution?. J Clin Oncol Off J Am Soc Clin Oncol.

[8] Eichler AF, Chung E, Kodack DP, Loeffler JS, Fukumura D, Jain RK (2011). The biology of brain metastases—translation to new therapies. Nat Rev Clin Oncol.

[9] Long GV, Trefzer U, Davies MA, Kefford RF, Ascierto PA, Chapman PB (2012). Dabrafenib in patients with Val600Glu or Val600Lys BRAF-mutant melanoma metastatic to the brain (BREAK-MB): a multicentre, open-label, phase 2 trial. Lancet Oncol.

[10] Margolin K, Ernstoff MS, Hamid O, Lawrence D, McDermott D, Puzanov I (2012). Ipilimumab in patients with melanoma and brain metastases: an open-label, phase 2 trial. Lancet Oncol..

[11] Long GV, Atkinson V, Menzies AM, Lo S, Guminski AD, Brown MP (2017). A randomized phase II study of nivolumab or nivolumab combined with ipilimumab in patients (pts) with melanoma brain metastases (mets): The Anti-PD1 Brain Collaboration (ABC). J Clin Oncol.

[12] Tio M, Wang X, Carlino MS, Shivalingam B, Fogarty GB, Guminski AD, et al. Survival and prognostic factors for patients with melanoma brain metastases in the era of modern systemic therapy. Pigment Cell Melanoma Res. 2018;31(4):509–15. 10.1111/pcmr.12682 29277979

[13] Gondi V, Pugh SL, Tome WA, Caine C, Corn B, Kanner A (2014). Preservation of memory with conformal avoidance of the hippocampal neural stem-cell compartment during whole-brain radiotherapy for brain metastases (RTOG 0933): a phase II multi-institutional trial. J Clin Oncol.

[14] Fogarty G, Morton RL, Vardy J, Nowak AK, Mandel C, Forder PM (2011). Whole brain radiotherapy after local treatment of brain metastases in melanoma patients--a randomised phase III trial. BMC Cancer.

[15] Mizumatsu S, Monje ML, Morhardt DR, Rola R, Palmer TD, Fike JR (2003). Extreme sensitivity of adult neurogenesis to low doses of X-irradiation. Cancer Res.

[16] Gondi V, Tome WA, Marsh J, Struck A, Ghia A, Turian JV (2010). Estimated risk of Perihippocampal disease progression after hippocampal avoidance during whole-brain radiotherapy: safety profile for RTOG 0933. Radiother Oncol J Eur Soc Ther Radiol Oncol..

[17] Hong AM, Suo C, Valenzuela M, Haydu LE, Jacobsen KD, Reisse CH (2014). Low incidence of melanoma brain metastasis in the hippocampus. Radiother Oncol J Eur Soc Ther Radiol Oncol.

[18] Sun B, Huang Z, Wu S, Shen G, Cha L, Meng X (2016). Incidence and relapse risk of intracranial metastases within the perihippocampal region in 314 patients with breast cancer. Radiother Oncol J Eur Soc Ther Radiol Oncol..

[19] Wang SJ, Choi M, Fuller CD, Salter BJ, Fuss M (2007). Intensity-modulated radiosurgery for patients with brain metastases: a mature outcomes analysis. Technol Cancer Res Treat.

[20] Lawson JD, Wang J-Z, Nath SK, Rice R, Pawlicki T, Mundt AJ (2010). Intracranial application of IMRT based radiosurgery to treat multiple or large irregular lesions and verification of infra-red frameless localization system. J Neuro-Oncol.

[21] Clark GM, Popple RA, Young PE, Fiveash JB (2010). Feasibility of single-isocenter volumetric modulated arc radiosurgery for treatment of multiple brain metastases. Int J Radiat Oncol Biol Phys.

[22] Gondi V, Cui Y, Mehta MP, Manfredi D, Xiao Y, Galvin JM (2015). Real-time pre-treatment review limits unacceptable deviations on a cooperative group radiotherapy technique trial: quality assurance results of RTOG 0933. Int J Radiat Oncol Biol Phys.

[23] Hippocampal contouring: a contouring Atlas for RTOG 0933. Available from: [https://www.rtog.org/CoreLab/ContouringAtlases/HippocampalSparing.aspx].

[24] Gondi V, Hermann BP, Mehta MP, Tomé WA. Hippocampal dosimetry predicts neurocognitive function impairment after fractionated stereotactic radiotherapy for benign or low-grade adult brain tumors. Int J Radiat Oncol Biol Phys. 2013;85(2):348–54. 10.1016/j.ijrobp.2012.11.031. 10.1016/j.ijrobp.2012.11.031 23312272

[25] Shen J, Bender E, Yaparpalvi R, Kuo H-C, Basavatia A, Hong L (2015). An efficient volumetric arc therapy treatment planning approach for hippocampal-avoidance whole-brain radiation therapy (HA-WBRT). Med Dosim.

[26] Pokhrel D, Sood S, McClinton C, Shen X, Lominska C, Saleh H (2016). Treatment planning strategy for whole-brain radiotherapy with hippocampal sparing and simultaneous integrated boost for multiple brain metastases using intensity-modulated arc therapy. Med Dosim Off J Am Assoc Med Dosim..

[27] Nevelsky A, Ieumwananonthachai N, Kaidar-Person O, Bar-Deroma R, Nasrallah H, Ben-Yosef R (2013). Hippocampal-sparing whole-brain radiotherapy using Elekta equipment. J Appl Clin Med Phys.

[28] Prokic V, Wiedenmann N, Fels F, Schmucker M, Nieder C, Grosu A-L (2013). Whole brain irradiation with hippocampal sparing and dose escalation on multiple brain metastases: a planning study on treatment concepts. Int J Radiat Oncol Biol Phys.

[29] Pokhrel D, Sood S, Lominska C, Kumar P, Badkul R, Jiang H (2015). Potential for reduced radiation-induced toxicity using intensity-modulated arc therapy for whole-brain radiotherapy with hippocampal sparing. J Appl Clin Med Phys.

[30] Wang S, Zheng D, Zhang C, Ma R, Bennion NR, Lei Y (2017). Automatic planning on hippocampal avoidance whole-brain radiotherapy. Med Dosim Off J Am Assoc Med Dosim.

[31] Sood S, Pokhrel D, McClinton C, Lominska C, Badkul R, Jiang H (2017). Volumetric-modulated arc therapy (VMAT) for whole brain radiotherapy: not only for hippocampal sparing, but also for reduction of dose to organs at risk. Med Dosim Off J Am Assoc Med Dosim..

[32] Cheah SK, Matthews T, Teh BS (2016). Hippocampal sparing whole brain radiotherapy and integrated simultaneous boost vs stereotactic radiosurgery boost: a comparative Dosimetric planning study. Asian Pac J Cancer Prev APJCP.

[33] Farjam R, Pramanik P, Aryal MP, Srinivasan A, Chapman CH, Tsien CI (2015). A radiation-induced hippocampal vascular injury surrogate marker predicts late neurocognitive dysfunction. Int J Radiat Oncol Biol Phys.

[34] Pospisil P, Kazda T, Bulik M, Dobiaskova M, Burkon P, Hynkova L, et al. Hippocampal proton MR spectroscopy as a novel approach in the assessment of radiation injury and the correlation to neurocognitive function impairment: initial experiences. Radiat Oncol. 2015;10:211. 10.1186/s13014-015-0518-1. 10.1186/s13014-015-0518-1 PMC4609038 26474857

[35] Pospisil P, Kazda T, Hynkova L, Bulik M, Dobiaskova M, Burkon P (2017). Post-WBRT cognitive impairment and hippocampal neuronal depletion measured by in vivo metabolic MR spectroscopy: results of prospective investigational study. Radiother Oncol J Eur Soc Ther Radiol Oncol..

[36] Kazda T, Vrzal M, Prochazka T, Dvoracek P, Burkon P, Pospisil P (2017). Left hippocampus sparing whole brain radiotherapy (WBRT): a planning study. Biomed Pap Med Fac Univ Palacky Olomouc Czechoslov.

[37] Lee TMC, Yip JTH, Jones-Gotman M (2002). Memory deficits after resection from left or right anterior temporal lobe in humans: a meta-analytic review. Epilepsia.

[38] Ma TM, Grimm J, McIntyre R, Anderson-Keightly H, Kleinberg LR, Hales RK (2017). A prospective evaluation of hippocampal radiation dose volume effects and memory deficits following cranial irradiation. Radiother Oncol.

[39] Gondi V, Hermann BP, Mehta MP, Tomé WA (2013). Hippocampal dosimetry predicts neurocognitive function impairment after fractionated stereotactic radiotherapy for benign or low-grade adult brain tumors. Int J Radiat Oncol Biol Phys.

[40] Tsai P-F, Yang C-C, Chuang C-C, Huang T-Y, Wu Y-M, Pai P-C, et al. Hippocampal dosimetry correlates with the change in neurocognitive function after hippocampal sparing during whole brain radiotherapy: a prospective study. Radiat Oncol. 2015;10:253 10.1186/s13014-015-0562-x PMC4676088 26654128

[41] Okoukoni C, McTyre ER, Ayala Peacock DN, Peiffer AM, Strowd R, Cramer C (2017). Hippocampal dose volume histogram predicts Hopkins verbal learning test scores after brain irradiation. Adv Radiat Oncol.

